# Supplementary material for: Region-specific expression of young small-scale duplications in the human central nervous system
Source: BMC Ecol Evol. 2021 Apr 21;21:59. doi: 10.1186/s12862-021-01794-w (PMC8059171; doi:10.1186/s12862-021-01794-w)
Supplement: Supplementary file 1 — Additional file 1. Supplementary information and figures. [file 12862_2021_1794_MOESM1_ESM.pdf]

**Result S1. Multivariate linear regression models to explain the Tau score**

*Confirmation of the effect of paralogy on the CNS region-specific expression*

Previous works have shown that a higher tissue-specificity is often associated with a lower expression level and that paralogs are less expressed on average than singletons (Guschanski et al. 2017). It is thus difficult to distinguish between the confounding effects of expression level and paralogy on the Tau score. Similarly, when looking at CNS regions, we observed that the maximal expression over all these regions was lower on average for a paralog than for a singleton, mostly due to SSDs (Figure 2), which could represent a confounding effect for the association that we found between region-specificity and paralogy.

In order to confirm the association of paralogy with CNS region-specific expression, in addition to the potential effect of expression level, we performed a multivariate linear regression using R version 3.5 (package stats). The model was fitted on all protein coding genes to predict Tau scores with explanatory variables corresponding to the maximal expression of each gene across the 7 CNS regions and its duplication status (singleton or paralog) (Additional File 2:Table S16). We observed that the maximal expression had a significantly negative effect on the Tau score, while the duplication status had a smaller but independent and significantly positive effect. The association between a high region-specificity and a low expression could be explained by two reasons: i) the calculation of the region-specificity Tau score can be artificially inflated for genes with low expression values in all regions, since expression ratios can be highly variable in the case of low discrete expression values (ie RPKM derived from low read counts), and ii) a low expression level can be related to region-specificity for some biological reasons. Thus, the same analysis was done on a more restricted set of genes by removing genes whose maximal expression is lower than 1 RPKM (instead of 0.1), in order to reduce the potential bias of low expressions. With the threshold of 1 RPKM, the effect of maximal expression was much less significant but the duplication effect was still significant and positive. These results indicate that

the specific expression of paralogous genes for a particular CNS region is influenced by both gene abundance value and duplication status.

*Confirmation of the effect of the age and the type of duplication on the region-specificity in the CNS*

Since SSDs (and in particular ySSDs) have a lower maximal expression than WGDs (Figure 2A), it is also difficult to distinguish between the confounding effects of expression and duplication type orage on Tau.

We then estimated the respective effects of the expression level (the maximal expression over CNS regions), the phyletic age of the duplication and the type of duplication (SSD or WGD) on Tau scores using multivariate linear regression models on paralogs only (Additional File 2: Table S16B). When using all paralogs with a maximal expression over CNS regions > 0.1 RPKM, we observed that the maximal expression had the stronger significant negative effect, while the duplication age had a weaker but still significant negative effect on region-specificity (the same results were obtained with the three age categories coded quantitatively: "0" for paralogs younger than WGD, "1" for WGD-old paralogs and "2" for paralogs older than WGD; Additional File 2: Table S16C). The duplication type effect did not appear since it is hidden by the duplication age effect, probably because WGDs are roughly all of the same age and constitute the very large majority of the paralogs of this age. We confirmed this hypothesis by applying the multivariate linear model on the subset of genes with the same age (WGD and wSSD) (Additional File 2: Table S16D). We found with this last model that indeed the SSD duplication type significantly contributed to a high region-specificity. Therefore, these results seem to confirm that ySSD genes tend to be more region-specific than other genes, due to both their SSD origin and their young duplication age, in addition to the effect of their low expression level.

As mentioned previously, the effect of expression on Tau score may be a true biological effect or a bias. We thus applied the same linear models after filtering out genes whose maximal expression over CNS regions was less than 1 RPKM, in order to reduce both the confounding effects and the risk of potential bias. We found that the effect of the duplication age was even

more significant than before this filter and that the effect of the duplication type became significant, while the effect of the maximal expression was really lower and less significant than before.

**Result S2. Association between region-specific expression in the same region and paralog pairs**

We analyzed the 130 paralog pairs for which both genes showed some region-specificity and with a single SSD or WGD annotation. We observed that, among these pairs, SSD pairs were slightly enriched in pairs where both paralogs were region-specific to the same region (50% of SSD pairs versus 31% of WGD pairs, p-value= 0.04483). The proportion of ySSD pairs that were region-specific to the same region was even higher (59%) but the enrichment was not significant, probably due to the very low number of ySSD pairs (22 pairs). This result suggests that within an SSD pair, especially a ySSD pair, with region-specific expression the two paralogs tend to be co-expressed.

**Result S3. Optimization of WGCNA parameters**

The WGCNA algorithm comprises five steps: 1) computation of a correlation measure for each gene pair to estimate the similarity of their expression profiles 2) computation of an adjacency matrix by applying a soft-threshold to the correlation measure (power applied to the correlation) 3) computation of the Topological Overlap Matrix (which reflects pairwise gene similarity in terms of connectivity with the other genes in the adjacency matrix), 4) hierarchical clustering of the Topological Overlap Matrix and 5) identification of modules of co-expressed genes from the hierarchical clustering.

*Soft-threshold.* The WGCNA tool generates the hypothesis that the majority of genes are weakly

connected by co-expression and only a small number of genes are highly connected. To respect this topology, known as scale-free, we had to find the appropriate soft threshold ($\beta$) implicated in the adjacency matrix computation (Equation 1). WGCNA tests different $\beta$ values and selects the one that most respects the scale-free topology. In this study, the soft threshold used was 6 (Figure S3A). For the adjacency matrix, we chose to use the equation that captures both the correlated and anti-correlated gene profiles, without distinction. Thus, for each gene pair (i,j) the following was defined:

(1) $A_{ij} = |\text{corPearson}|^{\beta}$

*Parameter selection.* One of our goals was to compare gene families to co-expression modules. Given that 47% of gene families have a size equal to 2, we optimized WGCNA parameters to obtain small highly co-expressed modules. Out of the collection of WGCNA parameters, we only evaluated those that impacted module size: the Cut tree, the deepSplit and the merge of clusters.

The Cut tree parameter corresponds to the height at which the hierarchical clustering tree is cut to obtain an intermediate number of clusters before splitting them into modules. Therefore, all the joining heights within a module must be below the height of the Cut Tree. The default value is 0.99. We tested different Cut tree values (from 0.95 to 0.99) (Figure S3B), and obtained the highest number of modules for the Cut tree values of 0.96 and 0.97. Greater values can lead to merging of some modules whereas smaller values can lead to removal of some modules (since their joining heights are then above the Cut Tree height). We chose a value between the two by looking at the size of the 20 largest modules (Figure S3C). These modules were smaller with a Cut tree value of 0.97.

Once the hierarchical tree is cut, initial clusters are subdivided into modules and the deepSplit parameter influences the module size by controlling the detection sensitivity of the modules. The higher the deepSplit value, the higher the detection sensitivity, resulting in more abundant and smaller modules. We assessed the size of the 20 largest modules for three different deepSplit

values (0, 2 and 4) (Figure S3D), and we obtained smaller modules with a value of 4 (highest value).

Finally to obtain small module sizes, we did not merge modules based on hierarchical clustering of their first principal components (called eigengenes).

**Result S4. Biological relevance of co-expression modules**

*Gene ontology and biological pathway analysis:* Gene ontology terms and biological pathways over-representation analyses were performed with the Panther classification system (released 2017-1205), using resources from the Gene Ontology database (release 2017-05-25) and the Reactome pathway database (Version 65 release 2018-06-12) (Fabregat et al. 2018). Gene ontology term over-representation in gene co-expression modules was performed with the GOStats package (Version 2.42.0) from Bioconductor (release 3.5).

*Enrichments of co-expression modules:* The biological relevance of our gene modules was determined using over-representation tests for gene ontology (GO) terms derived from molecular functions and biological processes. From the list of modules larger than 20 genes (81 modules), 67% and 86% respectively were found to be significantly enriched for at least one molecular function and one biological process (p-value threshold= 6.17E-04, after Bonferroni correction for the number of tested terms in each of these two categories, and the number of modules) (Additional File 2:Table S8).

**Result S5. Genomic distances between pairs of paralogs**

We then studied further the relationship between the type and categorical age of the duplication by analyzing the proximity between pairs of duplicates on the genome. We assessed the

association between duplicate proximity and each group of paralog pairs (SSD, ySSD, wSSD, oSSD and WGD), considering only the 2,918 paralog pairs with a single SSD or WGD annotation (Additional File 2: Table S20). We considered three categories of paralog pairs in terms of duplicate proximity: inter-chromosomal pairs (i.e. where each gene is on a different chromosome), intra-chromosomal pairs (i.e. both genes are on the same chromosome) and tandem duplicated pairs (i.e. both genes are on the same chromosome and separated by less than 1 Mb) (Lan and Pritchard 2016). As expected, we confirmed that the SSD pairs were depleted in the inter-chromosomal pair category (72.7% of SSDs versus 98.1% of non SSDs, p-value = 2.577E-44) and enriched in the tandem duplicates (21.6% of SSDs versus 0.1% of non SSDs, p-value = 2.014E-46). We obtained the same results for the ySSD pairs and observed that the proportion of tandem duplicate pairs was negatively associated with the categorical age of duplication (0% of oSSDs, 9.4% of wSSDs and 67.8% of ySSDs). Conversely, the proportion of inter-chromosomal pairs was positively associated with the age of the duplications (97.5% of oSSDs, 84.3% of wSSDs and 22.9% of ySSDs).

**Result S6. Characterization of homogeneous and heterogeneous gene families**

The comparison of the average size of families between each category showed that homogeneous families were significantly smaller than heterogeneous ones (Welch statistical test, average size of homogeneous families = 2.89, average size of heterogeneous families = 3.84, p-value = 8.278E-10). A total of 53 of these homogeneous families were completely included in the same module of co-expression. Furthermore, some modules were found to comprise several homogeneous gene families (Additional File 2: Table S9).

*Biological relevance*: A biological pathway enrichment analysis of the homogeneous family genes revealed that they were notably enriched in transcription factors and signaling proteins involved

in neural development (Additional File 2: Table S10): the AP-2 (TFAP2), HOX families of transcription factors and NOTCH signaling associated genes (Prince and Pickett 2002; Eckert et al. 2005).

In the families associated with heterogeneous expression of their member genes across CNS regions, we found enrichments in many signaling pathways, such as MET, NTRKs and MAPKs. We also identified the family of hyperpolarization-activated cation genes (HCN), which was previously known to be preferentially expressed in the brain (Santoro and Tibbs 1999; Calejo et al. 2014). We also identified significant enrichment in enzyme families involved in opioid signalling (adenyl cyclase, cyclic nucleotide phosphodiesterase) and glycosaminoglycan metabolism (glysosyltransferase) (Ludwig and Seuwen 2002; Breton et al. 2006; Halpin 2008).

The evolutionary constraints associated with the co-expression of genes within a family can highlight genes that work together to carry out their functions. On the opposite, families whose gene members are scattered over different co-expression modules could consist of genes which work independently and perhaps diverged in function through evolution. We found that these two categories of families differed in their level of enrichment in different biological processes and molecular functions. In homogeneous families, we showed that the majority of genes from the HOX and AP-2 families of transcription factors and from the NOTCH signaling pathway were strongly co-expressed.

**Result S7. Association between co-expression and shared region-specificity**

To first check the relationship between co-expression and shared region-specificity, we analyzed the distribution of region-specific genes across the 932 modules of co-expressed paralogs and found that 177 modules included at least two region-specific genes. We then looked at whether within each of these modules the region-specific genes were expressed in the same or in

different regions. We found that among these 177 modules, 66% consisted of region-specific genes associated with the same region (Additional File 2: Table S15). Therefore, gene modules identified from correlation-based co-expression networks also capture shared region-specificity.

**Result S8. Classification of human CNS regions from gene expression**

We focused specifically on CNS regions and assessed whether paralog expression could classify samples into regions. From gene expression profiles (filtered and adjusted RPKM values) generated by the GTEx Consortium for the 1,259 samples distributed across the 13 CNS regions, we did an unsupervised hierarchical clustering using the pheatmap package of R version 3.4 (similarity measure: Pearson correlation, clustering method: average linkage). This classification of human CNS samples, based on their pair-wise similarity in terms of correlation across paralog expression values, was able to group together most samples belonging to the same region (Methods; Additional File 1:Figure. S1). We observed a similar CNS region classification considering all protein-coding genes or only singletons (Additional File 1:Figure. S1).

**References:**

1. Guschanski K, Warnefors M, Kaessmann H. The evolution of duplicate gene expression in mammalian organs. Genome Res. 2017 Sep;27(9):1461–74.
2. Fabregat A, Jupe S, Matthews L, Sidiropoulos K, Gillespie M, Garapati P, Haw R, Jassal B, Korninger F, May B, Milacic M, Roca CD, Rothfels K, Sevilla C, Shamovsky V, Shorser S, Varusai T, Viteri G, Weiser J, Wu G, Stein L, Hermjakob H, D'Eustachio P. The Reactome Pathway Knowledgebase. Nucleic Acids Res. 2018 Jan 4;46(D1):D649-D655.
3. Lan X, Pritchard JK. Coregulation of tandem duplicate genes slows evolution of subfunctionalization in mammals. Science. 2016 May 20;352(6288):1009–13.
4. Prince VE, Pickett FB. Splitting pairs: the diverging fates of duplicated genes. Nat Rev Genet. 2002 Nov;3(11):827–37
5. Eckert, D., S. Buhl, S. Weber, R. Jager, and H. Schorle. 2005. The AP-2 family of transcription factors. Genome Biol. 6:246.
6. Gazave, E., Lapebie, P., Richards, G.S., Brunet, F., Ereskovsky, A.V., Degnan, B.M., Borchiellini, C.,Vervoort, M., Renard, E., 2009. Origin and evolution of the Notch signalling pathway: an

overview from eukaryotic genomes. BMC Evolutionary Biology 9, 249

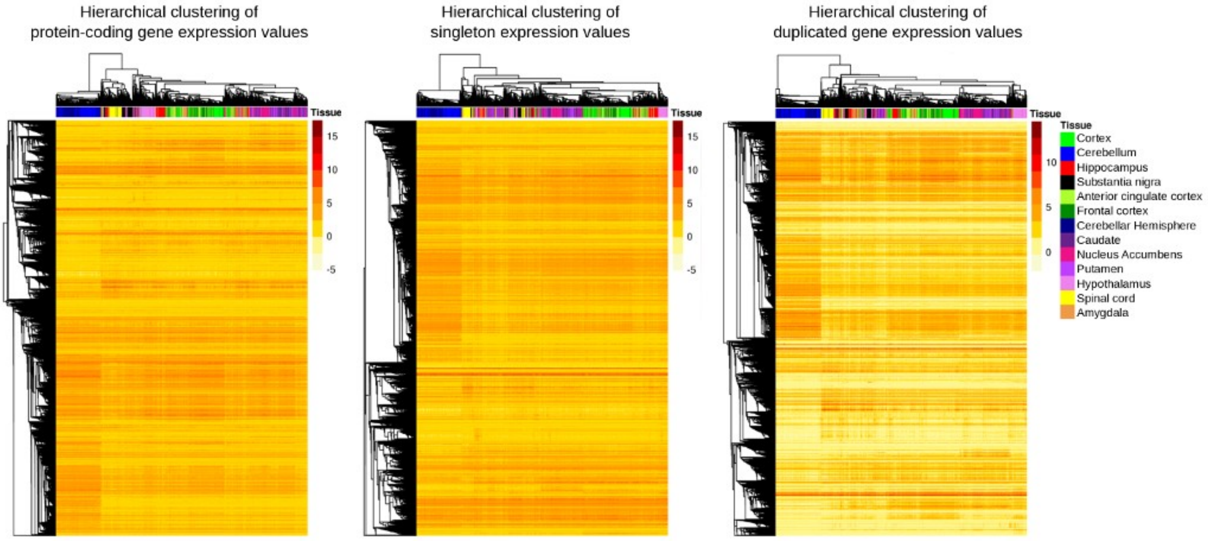


**Figure S1. Unsupervised hierarchical clustering of genes expressed in human central nervous system regions.** Hierarchical clustering of genes expressed in the CNS regions was performed based on gene pairwise distance in terms of correlation across gene expression values. The three gene groups considered are: protein-coding genes, singleton genes and paralogous genes. Each CNS region is represented by a different color. The regions belonging to the same anatomically defined CNS region are represented in the same color: blue for the cerebellum region (cerebellum and cerebellar hemisphere region), green for the cortex region (cortex, frontal cortex and anterior cingulate cortex regions), purple for the basal ganglia region (putamen, nucleus accumbens and caudate regions), and red for the amygdala-hippocampus region (amygdala and hippocampus regions). The remaining regions are considered as independent: pink for the hypothalamus region, yellow for the spinal cord region and black for the substantia nigra.

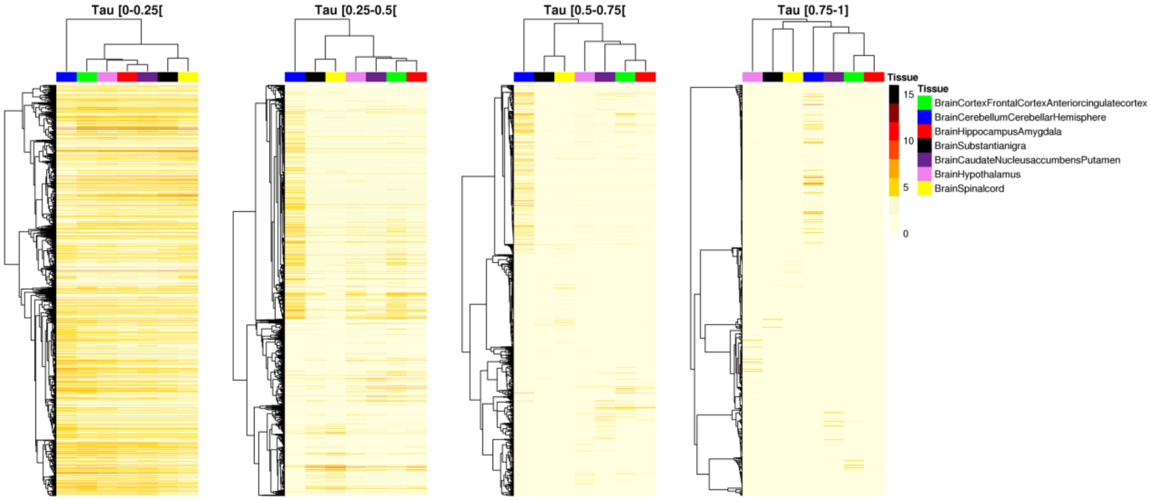


**Figure S2. Unsupervised hierarchical clustering of region-specific gene expression across CNS regions for different Tau score intervals.** Each heatmap illustrates mean gene expression across brain regions for a different Tau score interval.

**A**

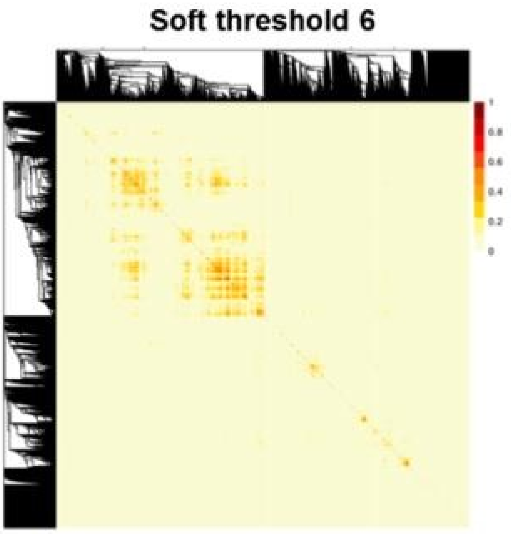
Soft threshold 6

**B**

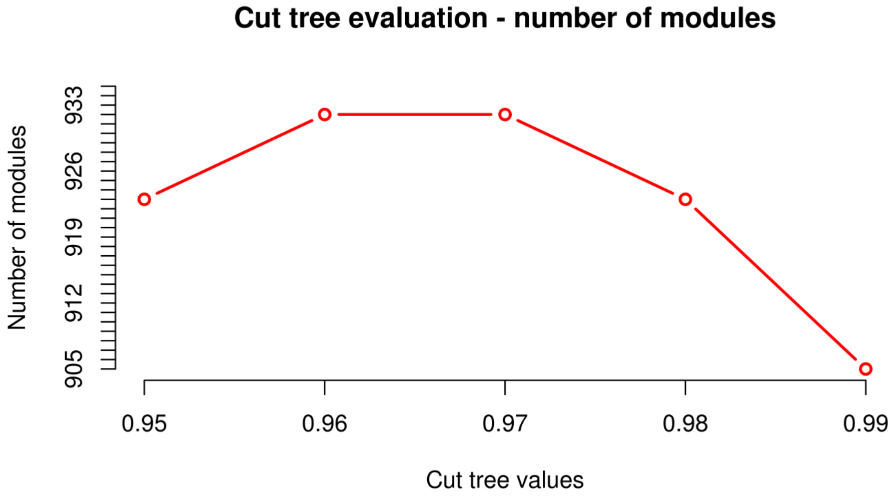
Cut tree evaluation - number of modules
Number of modules
905
912
919
926
933
0.95
0.96
0.97
0.98
0.99
Cut tree values

**C**

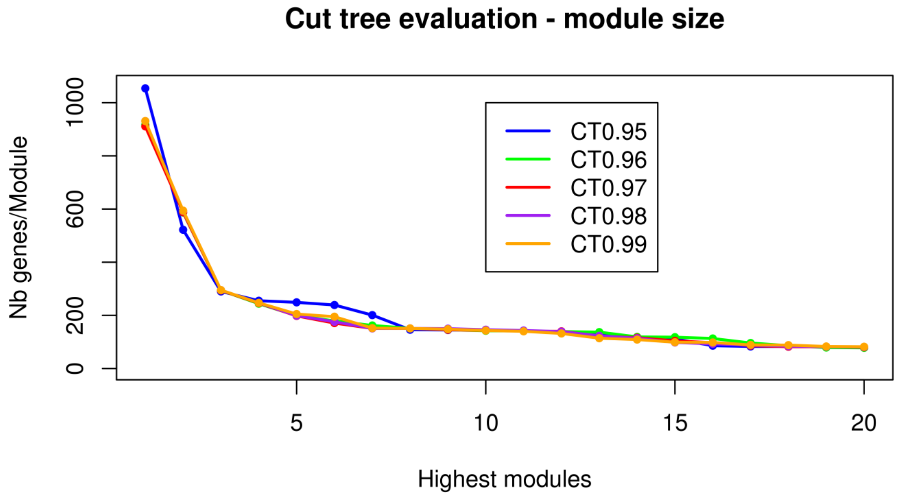
Cut tree evaluation - module size
Nb genes/Module
0
200
600
1000
5
10
15
20
Highest modules
CT0.95
CT0.96
CT0.97
CT0.98
CT0.99

**D**

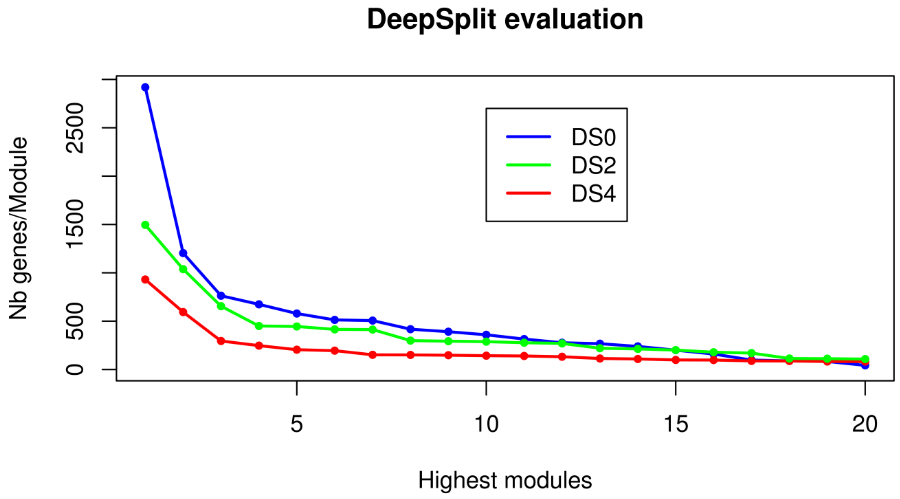


**Figure S3. Optimization of Weighted Gene Co-expression Network Analysis (WGCNA) parameters. (A)** Adjacency matrix for soft threshold parameter set to 6. **(B)** Iterative evaluation of Cut tree parameter on the generated number of co-expression modules. The x-axis represents different Cut tree values tested. **(C)** Iterative evaluation of Cut tree parameter on the generated co-expression module sizes. Color lines indicate tested Cut tree values: blue for 0.95, green for 0.96, red for 0.97, purple for 0.98 and orange for 0.99. **(D)** Iterative evaluation of DeepSplit parameter. Color lines indicate tested DeepSplit values: blue for 0, green for 2 and red for 4.

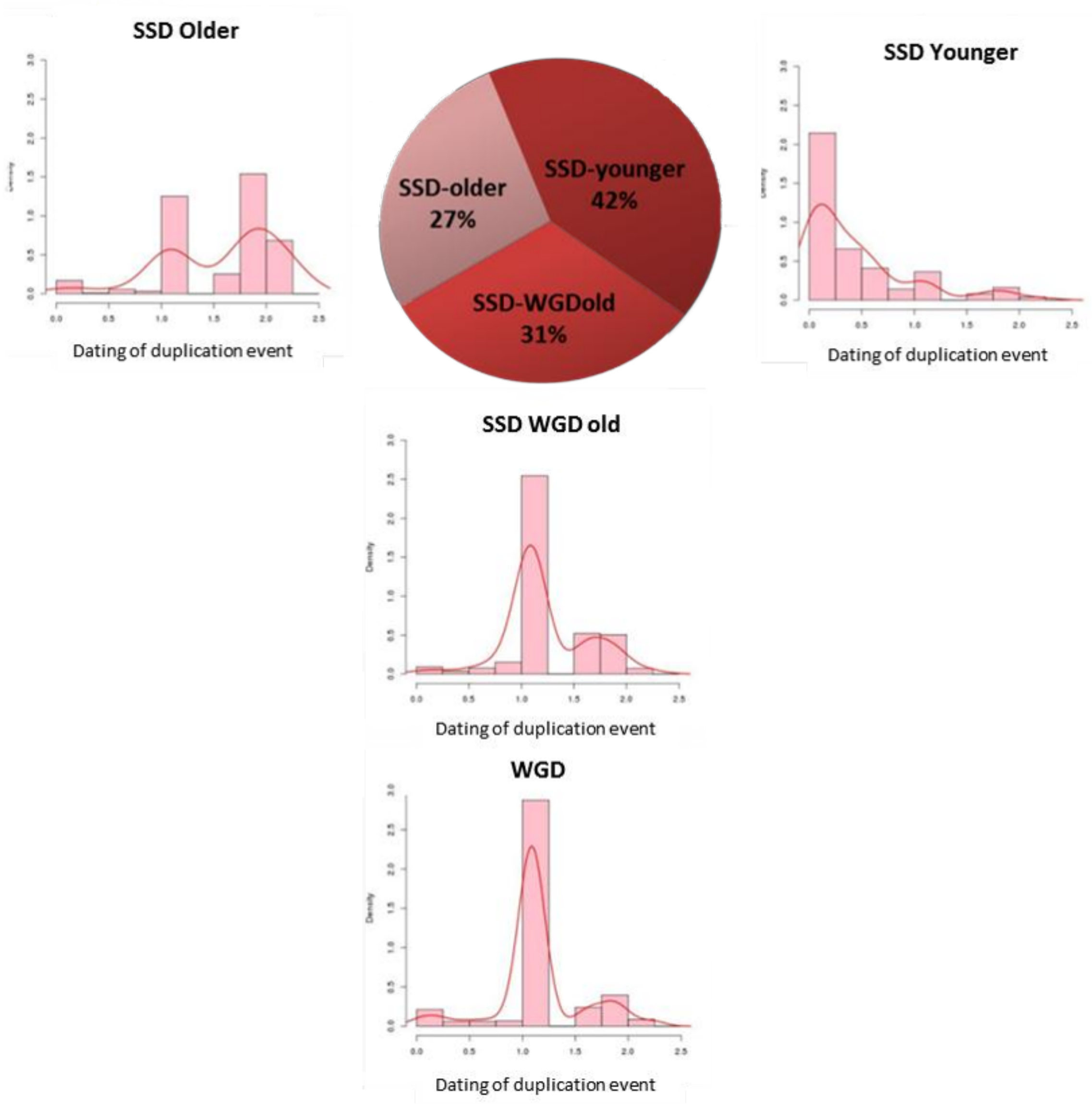


**Figure S4. Comparison between gene duplication dates generated by (Chen et al. 2013) and by (Singh et al. 2014).** For each gene duplication category defined by (Singh et al. 2014) an histogram generated using branch length from (Chen et al. 2013) is represented.

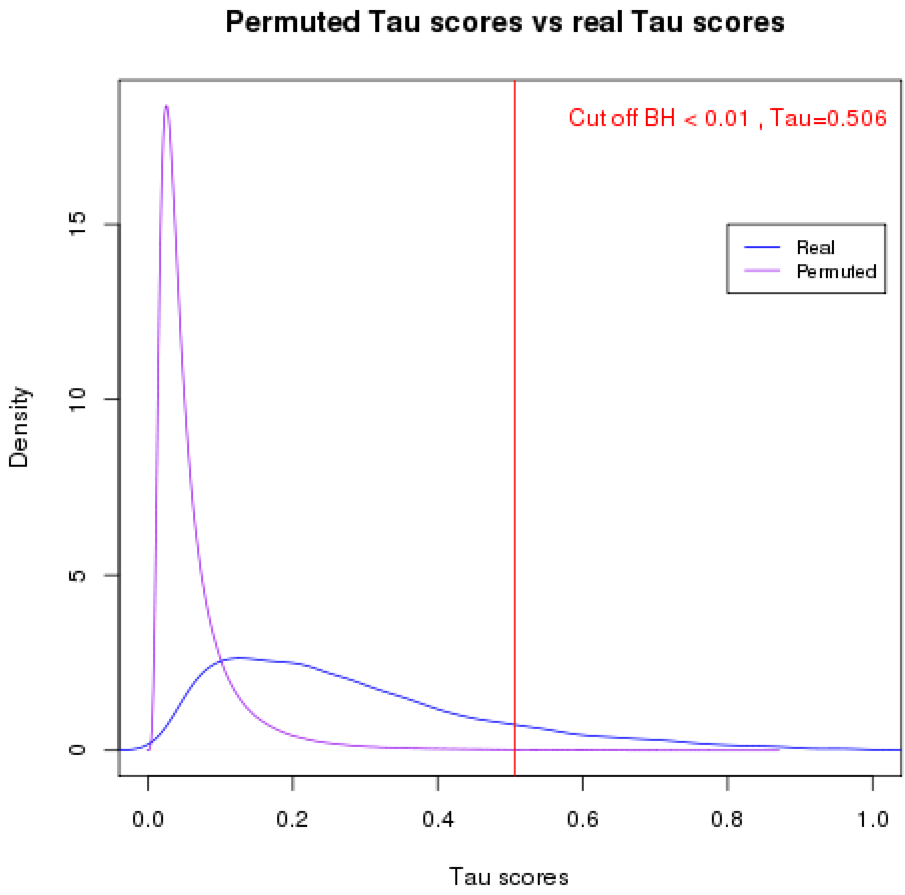


**Figure S5. Comparison between original and permuted Tau scores of protein coding genes across human CNS regions ( Expression threshold > 1 RPKM).**Density plot of original Tau scores (blue line) calculated from the expression values of 16427 protein coding genes, and permutated Tau scores (purple line) calculated from 1000 x 16427 permutations. The region specificity threshold of 0.506 (red dotted line) is defined, from permutated scores using the Benjamini-Hochberg corrected P-value of 0.01.
